# Proteomics analysis of differentially expressed proteins in chicken trachea and kidney after infection with the highly virulent and attenuated coronavirus infectious bronchitis virus *in vivo*

**DOI:** 10.1186/1477-5956-10-24

**Published:** 2012-03-31

**Authors:** Zhongzan Cao, Zongxi Han, Yuhao Shao, Xiaoli Liu, Junfeng Sun, Demin Yu, Xiangang Kong, Shengwang Liu

**Affiliations:** 1 Division of Avian Infectious Diseases, State Key Laboratory of Veterinary Biotechnology, Harbin Veterinary Research Institute, the Chinese Academy of Agricultural Sciences, Harbin 150001, People's Republic of China; 2 College of Animal Science and Veterinary Medicine, Shenyang Agricultural University, Shenyang 110866, People's Republic of China

**Keywords:** Infectious bronchitis virus, Proteomics, Chicken, Trachea, Kidney

## Abstract

**Background:**

Infectious bronchitis virus (IBV) is first to be discovered coronavirus which is probably endemic in all regions with intensive impact on poultry production. In this study, we used two-dimensional gel electrophoresis (2-DE) and two-dimensional fluorescence difference gel electrophoresis (2-DIGE), coupled with matrix-assisted laser desorption/ionization time-of-flight tandem mass spectrometry (MALDI-TOF/TOF-MS), to explore the global proteome profiles of trachea and kidney tissues from chicken at different stages infected *in vivo *with the highly virulent ck/CH/LDL/97I P_5 _strain of infectious bronchitis virus (IBV) and the embryo-passaged, attenuated ck/CH/LDL/97I P_115 _strain.

**Results:**

Fifty-eight differentially expressed proteins were identified. Results demonstrated that some proteins which had functions in cytoskeleton organization, anti-oxidative stress, and stress response, showed different change patterns in abundance from chicken infected with the highly virulent ck/CH/LDL/97I P_5 _strain and those given the embryo-passaged, attenuated P_115 _stain. In addition, the dynamic transcriptional alterations of 12 selected proteins were analyzed by the real-time RT-PCR, and western blot analysis confirmed the change in abundance of heat shock proteins (HSP) beta-1, annexin A2, and annexin A5.

**Conclusions:**

The proteomic alterations described here may suggest that these changes to protein expression correlate with IBV virus' virulence in chicken, hence provides valuable insights into the interactions of IBV with its host and may also assist with investigations of the pathogenesis of IBV and other coronavirus infections.

## Background

Coronaviruses (CoVs) are enveloped single-stranded positive sense RNA viruses that belong to the family *Coronaviridae *in the order *Nidovirales*. They are able to infect humans as well as other animals, including cows, pigs, mice, and chickens, they generally cause respiratory infection, gastrointestinal, and neurological disorders of varying severity. Infectious bronchitis virus (IBV) was the first coronavirus to be discovered, and is classed among the Gamma coronaviruses on the basis of antigenic and genetic relatedness [1]. It is a major poultry pathogen and is probably endemic in all chicken-raising regions; it has a severe impact on poultry production, causing heavy economic losses. All strains of IBV are capable of infecting a large range of epithelial surfaces of chickens, such as those of the trachea, kidney, oviduct and proventriculus [2].

Coronavirus infection has dramatic effects on host cell morphology, transcription and translation patterns, the cell cycle, cytoskeleton, suppression of interferon, and apoptosis pathways. Coronavirus infection may also cause inflammation, alter the immune and stress responses, and modify the coagulation pathways [3]. Such profound functional and morphological changes in host cells are associated with significant changes in the patterns of expression of host cell genes. Several studies have described changes in host gene expression associated with coronavirus infection, as documented by microarray technologies [4-9]. But ultimately, protein expression and post-translational modification (PTM) determine virus replication. Furthermore, transcriptome analyses only provide a snapshot of gene expression patterns; they also suffer from several limitations, including inconsistencies with the levels of expression of the corresponding proteins as well as lacking the ability to provide information on PTM. Approaches that use proteomics are promising because they can circumvent some of the issues associated with transcriptomics approaches [10]. More recently, comparative proteomics analysis has emerged as a valuable tool for the establishment of the global host protein profile in response to virus infection. It has been used to study enveloped RNA viruses such as influenza virus, respiratory syncytial virus (RSV), parainfluenza virus (PIV), human metapneumovirus (hMPV), SARS-CoV, and mouse hepatitis virus (MHV) [11-18]. It provides invaluable information on the cellular signaling pathways involved in either the cellular response to viral infections, or the viral manipulation of cellular machinery to ensure their own survival. For IBV, to the best of our knowledge, only some recent studies have investigated the changes in the expression of cellular proteins during IBV infection *in ex vivo *or *in ovo *[19-21]. However, the *in vivo *infection model could yield more biologically relevant insights into pathogenesis.

In this study, we used two-dimensional gel electrophoresis (2-DE) and two-dimensional fluorescence difference gel electrophoresis (2-DIGE), coupled with matrix-assisted laser desorption/ionization time-of-flight tandem mass spectrometry (MALDI-TOF/TOF-MS), to explore global changed proteome profiles of trachea and kidney tissues from chicken at different stages infected *in vivo *with the highly virulent ck/CH/LDL/97I P_5 _strain of IBV and an embryo-passaged strain of attenuated virulence, ck/CH/LDL/97I P_115_. In total, 58 differentially expressed proteins were identified and classified into several functional categories, including cytoskeleton organization, anti-oxidative stress, the stress response, acute phase response, and energy metabolism. In addition, the dynamic transcriptional alterations of 12 selected proteins were analyzed by the real-time RT-PCR method. Simultaneously, western blot analysis confirmed the change in abundance of the heat shock proteins (HSP) beta-1, annexin A2, and annexin A5. The potential roles of some of these identified proteins are discussed in order to characterize their potential functional roles during IBV infection *in vivo*. These results provide valuable insights into the interactions of IBV with its host, and may also be useful in investigations of the pathogenesis of IBV and other coronaviruses.

## Results

### IBV antibody detection and observed clinical signs

All chickens exhibited respiratory clinical signs at about 4-14 dpi with the IBV ck/CH/LDL/97I P_5 _strain. The clinical signs included tracheal rales, watery eyes, nasal mucus, and sneezing. The clinical signs shown by the inoculated birds tended to disappear gradually after 14 dpi. Gross lesions of the chickens killed in the P_5_-infected group were confined mainly to the kidneys. The kidney parenchyma of the dead birds was pale, swollen and mottled; the tubules and urethras were distended with uric acid crystals [22]. For chickens in the control group and those inoculated with the IBV ck/CH/LDL/97I P_115 _strain, no respiratory clinical signs and no gross lesions were observed during the experimental period.

As summarized in Additional file 1, no chickens inoculated with the ck/CH/LDL/97I P_5 _or the ck/CH/LDL/97I P_115 _strain of IBV showed seroconversion at 4 dpi. Antibodies appeared at 7 dpi and all of the chickens showed seroconversion after 14 dpi with both IBV strains. The chickens in the non-inoculated control group showed a negative serum antibody response.

### Analysis of viral load in the trachea and kidney of IBV-infected chickens

**Figure 1:**
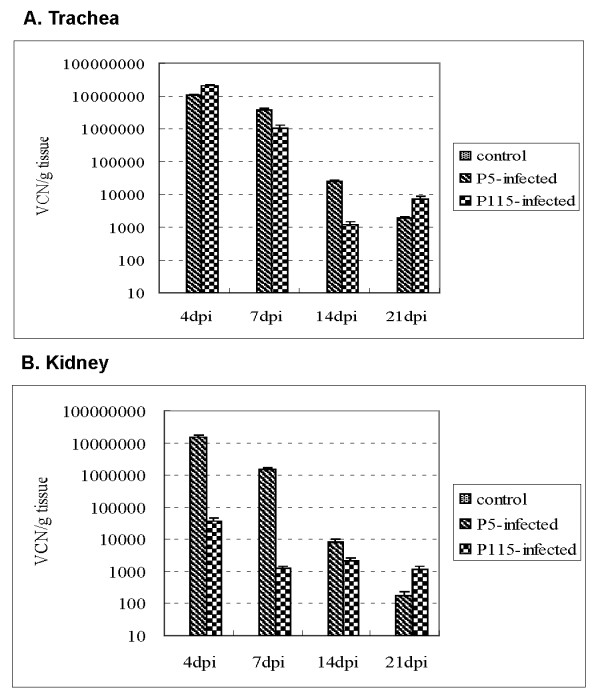
**The viral load in each sample was quantified using real-time RT-PCR**. The average viral copy number (VCN) per g tissue of each group was calculated. Error bars indicate standard error of the mean, and dpi represent days post-inoculation.

Successful IBV infection was also verified using real-time RT-PCR. Results are presented in Figure 1. Virus was not detected from the trachea and kidney of chickens from control group. In the P_5_-infected group, virus was detected from trachea and kidney at 4, 7, 14, and 21 dpi, the peak of viral copy number was reached at 4 dpi, after which time viral load fell. In trachea of P_115_-infected group, the peak of viral copy number was at 4 dpi, then fell until 14 dpi, and a little fluctuated at 21 dpi. In kidney of P_115_-infected group, the peak of viral copy number was also at 4 dpi, then fell at 7 dpi, and only a little fluctuated at 14 and 21 dpi. Furthermore, both in trachea and kidney, the P_115_-infected group had the lower viral genome copies than P_5_-infected group.

### Analysis of differentially changed proteins in abundance by 2-DE and 2-DIGE

**Figure 2:**
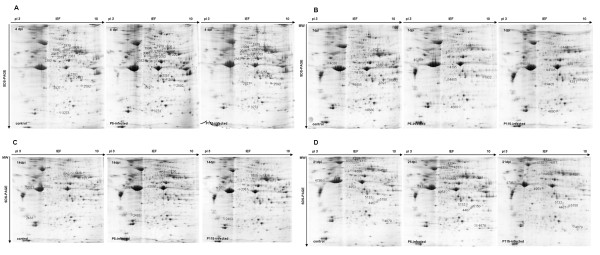
**Representative 2-DE maps of tracheal tissues from the IBV ck/CH/LDL/97I P_5_-infected group, P_115_-infected group and control group**. (A) 4 dpi; (B) 7 dpi; (C) 14 dpi; (D) 21 dpi. Protein samples were separated on 13 cm pH 3-10 linear IPG strips, followed by SDS-PAGE, and stained with Coomassie Blue R-350. The images were analyzed using Image Master 2D Platinum 6.0 software. The differentially expressed protein spots identified were marked with circles and labeled with the respective Match ID listed in Tables 1 and 2.

**Figure 3:**
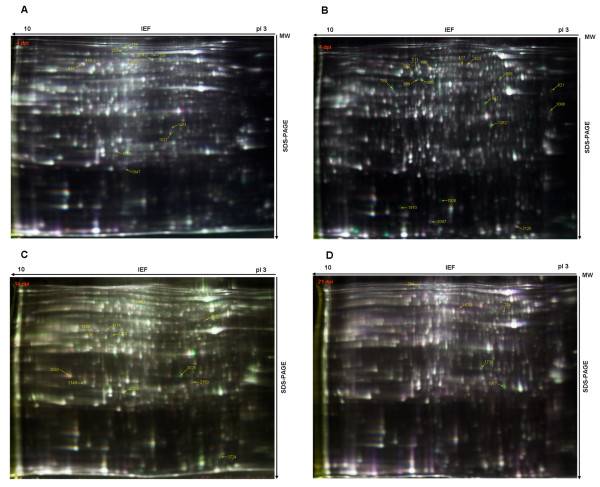
**Representative 2-DIGE maps of kidney tissues from the IBV ck/CH/LDL/97I P_5_-infected group, P_115_-infected group and control group**. (A) 4 dpi; (B) 7 dpi; (C) 14 dpi; (D) 21 dpi. Protein samples labeled with fluor were separated on 24 cm pH 3-10 linear IPG strips, followed by SDS-PAGE, gels were scanned on a Typhoon 9400 scanner, and image analysis was performed with Ettan™ DeCyder Software version v6.5. The protein spots identified were marked with circles and labeled with the respective Match ID listed in Tables 3 and 4.

The global protein changes in the chicken trachea and kidney tissues at different stages after infection with the IBV ck/CH/LDL/97I P_5 _and ck/CH/LDL/97I P_115 _strain were investigated. For the tracheal protein samples, Figure 2 shows representative images of the tracheal samples: 1366 ± 39, 1536 ± 126, and 1600 ± 167 protein spots were detected in gels from the control group, P_5_-infected group, and P_115_-infected group at 4 dpi; 1355 ± 300, 1518 ± 175, and 1078 ± 122 protein spots were detected in gels from the control group, P_5_-infected group, and P_115_-infected group at 7 dpi; 1293 ± 91, 1365 ± 126, and 1220 ± 56 protein spots were detected in gels from the control group, P_5_-infected group, and P_115_-infected group at 14 dpi; 1204 ± 91, 1236 ± 42, and 1111 ± 50 protein spots were detected in gels from the control group, P_5_-infected group, and P_115_-infected group at 21 dpi. For the kidney protein samples, Figure 3 shows representative images of the kidney samples at 4, 7, 14, and 21 dpi: 2315 ± 87, 2482 ± 189, 2607 ± 238, and 2593 ± 192 protein spots were detected, respectively.

The number of differentially expressed protein spots in the P_5 _and P_115 _infected groups is summarized in Additional file 2. In the tracheal total proteome at 4 dpi, the host response to infection with both P_5 _and P_115 _strains appears to involve predominantly increase of proteins abundance. By 7 dpi, the pattern is different, with infection with the P_115 _strain resulting in a predominant increase of proteins abundance, and with P_5 _exhibiting a majority of decrease events. By 14 and 21 dpi, both viruses produce a broadly similar response, with the vast majority of changes in protein abundance involving increase. For the kidney total proteome, at 4 dpi, P_5 _infection induced an approximately equivalent number of proteins with increased and decreased abundance, whereas P_115 _infection induced mainly increase of proteins abundance. By 7, 14, and 21 dpi, both P_5 _and P_115 _infection induced mainly increase of host proteins abundance.

### Identification of differentially changed proteins in abundance by MS

All interesting changed protein spots in trachea and kidney tissues were excised, and identified by MALDI-TOF-TOF MS. From trachea and kidney tissues, 24 and 34 proteins were successfully identified, respectively. Detailed information on the identified proteins is provided in Tables 1, 2, 3 and 4, Additional file 3, and Additional file 4. Figures 2 and 3 show representative gels indicating protein spots identified in tracheal and kidney tissues respectively. As shown in Tables and Figures, several proteins were identified in multiple spots with the same molecular weight but different isoelectric points, such as three spots contained lamin-A (spot # 4317, 4310, 4321 in Figure 2).

**Table 1:** Similar abundance changed proteins in tracheal from chicken infected with IBV ck/CH/LDL/97I P_5 _and ck/CH/LDL/97I P_115_

Match ID^a^	Protein description	Accession no.^b^	Protein score	Protein score CI%^c^	Mw (Da)	p*I*	Average ratio and *p*-value															
							
							P_5_-infected/control								P_115_-infected/control							
							
							4 dpi		7 dpi		14 dpi		21 dpi		4 dpi		7 dpi		14 dpi		21 dpi	
							
							ratio	*p*	ratio	*p*	ratio	*p*	ratio	*p*	ratio	*p*	ratio	*p*	ratio	*p*	ratio	*p*
4467	annexin A1 (ANXA1)	gi|46195459	910	100	38761	7.05							0.89	0.435							0.49	0.001
2051	B-creatine kinase	gi|211235	631	100	42525.5	5.78					1.44	0.025							1.77	0.006		
1926, 1736, 1817, 4581	cartilage matrix protein precursor	gi|71896317	314	100	54798.1	6.63	2.71	0.006			1.24	0.427	1.94	0.003	2.48	0.025			1.96	0.012	2.46	0
1987	fibrinogen beta chain	gi|211780	612	100	53271.8	7.18	2.75	0.006							2.57	0.011						
4679	glutathione S-transferase class-alpha	gi|4959550	79	99.958	21326.5	8.03							1.36	0.005							1.8	0
4036	keratin, type I cytoskeletal 19	gi|45384356	772	100	46111	4.94			2.43	0.016							2.15	0.009				
5150	nmrA-like family domain-containing protein 1	gi|71897147	653	100	32726	7.77							2.21	0.004							1.72	0.009
4405	PREDICTED: microfibril-associated glycoprotein 4 (EST)	gi|18470900	331	100	28600	5.2			0.57	0.001							0.97	0.695				
2021	Vimentin (VIM)	gi|212868	1160	100	53166.6	5.09					0.76	0.015							0.56	0.001		

a) Match ID represents unique numbers assigned to each spot in the matched standard of ImageMaster 2D Platinum version 6.0 software

b) Accession no. is the MASCOT result of MALDI-TOF/TOF searched from the NCBInr database

c) The criterion for a successfully identified protein is a protein score confidence interval (C.I. %) for PMF and MS/MS data ≥ 95%

**Table 2:** Differential abundance changed proteins in tracheal from chicken infected with IBV ck/CH/LDL/97I P_5 _and ck/CH/LDL/97I P_115_

Match ID^a^	Protein description	Accession no.^b^	Protein score	Protein score CI%^c^	Mw (Da)	p*I*	Average ratio and *p*-value															
							
							P_5_-infected/control								P_115_-infected/control							
							
							4 dpi		7 dpi		14 dpi		21 dpi		4 dpi		7 dpi		14 dpi		21 dpi	
							
							ratio	*p*	ratio	*p*	ratio	*p*	ratio	*p*	ratio	*p*	ratio	*p*	ratio	*p*	ratio	*p*
2079, 2083	aldehyde dehydrogenase 2 family	gi|118098552	983	100	64491.8	8.79	1.55	0.001							2.72	0.09						
2751	aldo-keto reductase	gi|14330324	795	100	36790.8	7.63	0.99	0.05							2.48	0.079						
2382	alpha-enolase	gi|46048768	611	100	47617.5	6.17	4.87	0.019							2.28	0.91						
2481, 4391	annexin A2 (ANXA2)	gi|45382533	783	100	38901	6.92	1.54	0.371	N/A	0					2.44	0.012	4.63	0.003				
2224	cartilage matrix protein precursor	gi|71896317	1090	100	54798.1	6.63	12.82	0.019							2.59	0						
1689	creatine kinase M-type	gi|211528	443	100	43529.1	6.5			0.87	0.567							5.63	0.001				
4195, 4953	fructose-bisphosphate aldolase C	gi|999392	256	100	39022.8	5.79			0.34	0.009			0.26	0			2.25	0.009			2.23	0.002
3273	heat shock protein beta-1 (HSPB1)	gi|45384222	766	100	21715	5.77	5.42	0.005							2.66	0.955						
4317 4310, 4321	lamin-A	gi|45384214	651	100	73348.5	6.5							2.88	0							0.41	0.001
2592	nmrA-like family domain-containing protein 1	gi|71897147	1090	100	32726	7.77	0.56	0.001							2.78	0.003						
2483	similar to D4-GDP-dissociation inhibitor (GDID4)	gi|50728568	552	100	22928.6	5.08					2.37	0.039							1.61	0.254		
1932	similar to malate dehydrogenase 2, NAD (mitochondrial)	gi|50758110	819	100	37400.6	8.83			N/A	0.019							1.3	0.31				
5132	similar to myozenin	gi|50749396	319	100	29589.7	7.85							2.24	0.058							N/A	
1919, 1659	pyruvate kinase (PKM2)	gi|212571	291	100	58433.9	7.29	1.12	0.488	0.44	0.071	0.5	0.012			2.55	0.004	2.81	0.024	1.28	0.088		
4635	selenium binding protein 1	gi|118102241	731	100	53097.6	6.17							0.82	0.156							1.86	0.034
2016	succinyl-CoA:3-ketoacid-coenzyme A transferase 1, mitochondrial	gi|60592998	201	100	56549.4	8.01	5.49	0.138							2.09	0						
4680	UMP-CMP kinase	gi|71896025	947	100	22386.3	6.75			1.07	0.56							2.42	0.002				
4786	vimentin (VIM)	gi|212868	1210	100	53166.6	5.09							4.78	0							1.4	0.394

a) Match ID represents unique numbers assigned to each spot in the matched standard of ImageMaster 2D Platinum version 6.0 software

b) Accession no. is the MASCOT result of MALDI-TOF/TOF searched from the NCBInr database

c) The criterion for a successfully identified protein is a protein score confidence interval (C.I. %) for PMF and MS/MS data ≥ 95%

N/A: A indicates the spot was detectable on one of the gels, N indicates the spot was too weak to detect on one of the gels

**Table 3:** Similar abundance changed proteins in kidney from chicken infected with IBV ck/CH/LDL/97I P_5 _and ck/CH/LDL/97I P_115_

Master ID^a^	Protein description	Accession no.^b^	Protein score	Protein score CI%^c^	Mw (Da)	p*I*	Average ratio and *p*-value															
							
							P_5_-infected/control								P_115_-infected/control							
							
							4 dpi		7 dpi		14 dpi		21 dpi		4 dpi		7 dpi		14 dpi		21 dpi	
							
							ratio	*p*	ratio	*p*	ratio	*p*	ratio	*p*	ratio	*p*	ratio	*p*	ratio	*p*	ratio	*p*
2033	carbonyl reductase [NADPH] 1	gi|71895267	1110	100	30519.9	8.5													1.66	0.005		
435	catalase	gi|53127216	529	100	60279	8.09	0.64	0.002							0.67	0						
252, 264	chain A, the structure of chicken mitochondrial Pepck in complex with Pep (PCK2)	gi|158430534	989	100	68009.9	6.55	4.27	0.015							3.6	0.019					1.56	0.038
2125, 3724	chain A, transthyretin	gi|1633502	672	100	14209	5.1			0.63	0.005	0.99	0.88					0.83	0.065	0.64	0		
658	chain C, crystal structure of native chicken fibrinogen with two different bound ligands	gi|21730885	659	100	47400.8	5.32			1.71	0.039							1.68	0.022				
1306	class I alcohol dehydrogenase, beta subunit	gi|45384164	286	100	40891.8	7.85					1.49	0.001							1.54	0.001		
2087	cytochrome c oxidase subunit 4 isoform 1, mitochondrial	gi|71895513	359	100	19676.1	8.91			0.58	0.008							0.57	0.026				
1274	D-amino-acid oxidase	gi|118098567	298	100	41209.8	6.7					1.4	0.15							1.55	0.001		
144	ovotransferrin BB type	gi|71274075	1020	100	79606.1	6.85	1.3	0.003							1.56	0						
374	similar to CDNA sequence BC048390	gi|118098312	1030	100	65576.4	7.85	0.64	0.001							0.60	0						
417	similar to CDNA sequence BC048390	gi|118098312	734	100	65576.4	7.85			0.52	0.008							0.81	0.13				
422	similar to CDNA sequence BC048391	gi|118098312	865	100	65576.4	7.85			1.35	0.033							1.52	0.021				
436	similar to cystathionase	gi|118094764	618	100	44554.7	6.86	0.78	0.032							0.66	0.001						
2149	similar to dodecenoyl-coenzyme A delta isomerase (3,2 trans-enoyl-coenzyme A isomerase)	gi|118098151	502	100	34557.8	9.3					1.63	0.003							1.19	0.023		
670	similar to FLJ20699 protein	gi|118083181	352	100	52820.8	5.95							0.75	0.006							0.5	0
1527, 2150	similar to guanidinoacetate N-methyltransferase	gi|118103242	424	100	17457.7	6.59	0.63	0			0.65	0.001			0.73	0.001			0.86	0.002		
1174	receptor-associated protein	gi|2661436	440	100	40792.2	8.61					1.66	0.032							1.28	0.11		
511	retinal dehydrogenase 1	gi|45383031	534	100	56400.8	7.49			1.26	0.096							1.57	0.013				
1739	similar to methyltransferase 24	gi|50750103	614	100	30640.5	6							1.65	0							1.87	0

a) Match ID represents unique numbers assigned to each spot in the matched standard of Ettan™ DeCyder 2D v6.5 software

b) Accession no. is the MASCOT result of MALDI-TOF/TOF searched from the NCBInr database

c) The criterion for successful identification of a protein is a protein score confidence interval (C.I. %) for PMF and MS/MS data ≥ 95%

**Table 4:** Differential abundance changed proteins in kidney from chicken infected with IBV ck/CH/LDL/97I P_5 _and ck/CH/LDL/97I P_115_

Master ID^a^	Protein description	Accession no.^b^	Protein score	Protein score CI%^c^	Mw (Da)	p*I*	Average ratio and *p*-value															
							
							P_5_-infected/control								P_115_-infected/control							
							
							4 dpi		7 dpi		14 dpi		21 dpi		4 dpi		7 dpi		14 dpi		21 dpi	
							
							ratio	*p*	ratio	*p*	ratio	*p*	ratio	*p*	ratio	*p*	ratio	*p*	ratio	*p*	ratio	*p*
496	aldehyde dehydrogenase 4 family, member A1	gi|118101121	1010	100	112936	5.4			0.56	0.009							0.85	0.031				
662	aldehyde dehydrogenase 4 family, member A1	gi|118101121	890	100	112936	5.4					1.03	0.84							1.59	0.015		
452	alpha-enolase	gi|46048768	353	100	47617.5	6.17	1.25	0.14							1.7	0.003						
821, 1088	alpha-tropomyosin of smooth muscle (TPM1)	gi|833618	94	99.999	32962.7	4.67			2.22	0.003							1.16	0.46				
1977	apolipoprotein A-I (APOA1)	gi|211159	703	100	30673.2	5.58							0.97	0.31							1.58	0
1262	chain A, crystal structures of chicken annexin V in complex with Ca^2+ ^(ANXA5)	gi|62738641	728	100	36158.6	5.61			2.04	0.004							1.1	0.75				
983, 784	chain C, crystal structure of native chicken fibrinogen	gi|8569623	921	100	47485.9	5.4					1.69	0.002	1.24	0.034					1.07	0.35	0.76	0.006
1926	low molecular weight phosphotyrosine proteinphosphatase (TCP1)	gi|86129490	304	100	18640.2	6.81			6.34	0.045							0.74	0.16				
1847	manganese-containing superoxide dismutase precursor (MNSOD)	gi|12034955	258	100	25158.7	8.6	1.52	0.015							1.12	0.022						
1970	nucleoside diphosphate kinase	gi|2827446	445	100	17542	7.11			4.68	0.003							1.48	0.81				
293	phosphoenolpyruvate carboxykinase (EC 4.1.1.32)	gi|212538	492	100	70224.3	6.1	1.15	0.047							1.78	0						
665	similar to aflatoxin aldehyde reductase	gi|118101125	286	100	36943.4	6.76			2.61	0.004							1.28	0.059				
789	similar to betaine homocysteine methyltransferase	gi|50755288	1010	100	45552.1	7.56			2.51								1.41					
582	glutamate dehydrogenase 1, mitochondrial	gi|118534	339	100	56075.4	8.48			1.74	0.051							1.06	0.17				
1461, 2026	similar to methyltransferase 24 (MET24)	gi|50750103	556	100	30640.5	6	0.72	0.002			1.25	0.074			1.09	0.066			0.42	0		
987	sulfotransferase	gi|45384226	265	100	36333.5	5.89			4.53	0.01							0.98	0.85				
1650	triosephosphate isomerase (EC 5.3.1.1)	gi|212774	1290	100	26831.9	6.71	1.51	0.009							1.07	0.43						

a) Match ID represents unique numbers assigned to each spot in the matched standard of Ettan™ DeCyder 2D v6.5 software

b) Accession no. is the MASCOT result of MALDI-TOF/TOF searched from the NCBInr database

c) The criterion for successful identification of a protein is a protein score confidence interval (C.I. %) for PMF and MS/MS data ≥ 95%

According to annotations from the UniProt Knowledgebase (UniProtKB) and the Gene Ontology databases, most of the identified proteins were involved in cytoskeleton organization, anti-oxidative stress, the stress response, acute phase response, energy metabolism, macromolecular biosynthesis, signal transduction and ion transport (summarized in Additional file 5). Among them, the abundance of annexin A2, annexin A5, pyruvate kinase (PKM2), alpha-enolase, mitochondrial phosphoenolpyruvate carboxykinase (PCK2), triosephosphate isomerase, heat shock protein beta-1, manganese-containing superoxide dismutase (MnSOD), vimentin, lamin-A, cartilage matrix protein, alpha-tropomyosin, nucleoside diphosphate kinase, sulfotransferase, and low molecular weight phosphotyrosine proteinphosphatase, were induced to be differentially patterns changed in chickens infected with the P_5 _and P_115 _strains, suggested that infection with the P_5 _and P_115 _strains produces different host response. Furthermore, this difference in the pattern of change was induced predominantly in the early stages of the infection cycle, which suggests that critical events early in infection are likely to be of key importance in determining the fate of the host. In addition, fibrinogen β and γ chains showed a similar pattern of change, with increase in the P_5_- and P_115_-infected groups.

### Gene ontology annotations of differentially changed proteins in abundance

**Figure 4:**
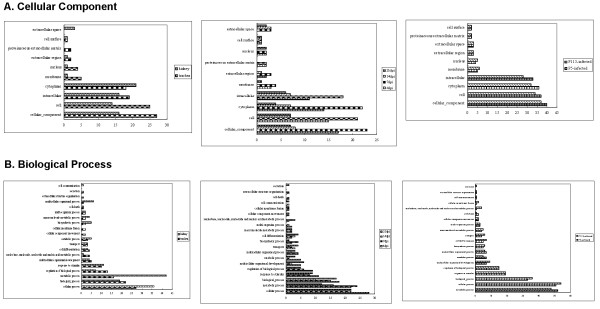
**Gene Ontology annotation analysis of differentially expressed proteins according to their cellular component (A) and biological process (B)**. This classification was produced on the basis of an analysis using the *GOSlimViewer *tool at the Agbase database http://www.agbase.msstate.edu/. The results were compared between trachea and kidney, P5-infected and P115-infected group, and different day's post-inoculation.

In order to generate an overview of the subcellular location and biological processes of this abundance changed proteins in trachea and kidney, P_5_-infected and P_115_-infected group, and different dpi, categorization of these proteins was performed on the basis of Gene Ontology (GO) annotations. As shown in Figure 4A, cellular component ontology revealed that the majority of the identified proteins were associated with intracellular (GO:0005622) and cytoplasm (GO:0005737) both in trachea and kidney, P_5_-infected and P_115_-infected group, and different dpi. As shown in Figure 4B, biological process ontology revealed that the majority of the identified proteins were associated with metabolic processes (GO:0008152), response to stimulus (GO:0050896), and regulation of biological process (GO:0050789) both in trachea and kidney, P_5_-infected and P_115_-infected group, and different dpi.

### Analysis of mRNA level by real-time RT-PCR

**Figure 5:**
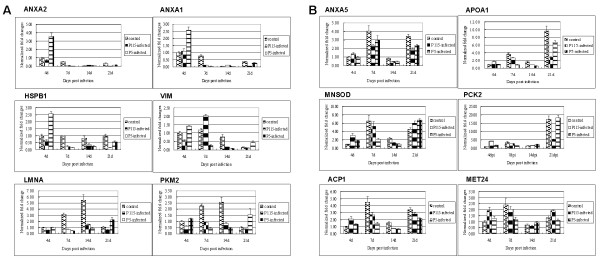
**Transcript analysis of the 12 proteins differentially expressed in trachea (A) and kidney (B) of chickens infected with the IBV ck/CH/LDL/97I P_5 _and ck/CH/LDL/97I P_115 _strains by real-time RT-PCR**. Total RNA extracts were prepared from the trachea and kidney of chickens in the control, P_5_-infected, and P_115_-infected group's at all four time points. Data represent means of three biological replicates per group. Error bars indicate standard error. Samples were normalized with the expression of the 18S ribosomal RNA gene. For symbols indicating different genes, refer to Tables 1, 2, 3, and 4.

The alterations in the mRNA level of 12 selected proteins in the trachea or kidney from the control, P_5_-infected, and P_115_-infected groups were analyzed at 4, 7, 14, and 21 dpi. For genes from tracheal tissue, as shown in Figure 5A, at 4 dpi, the mRNA level was up-regulated in the P_5_-infected group, when compared with the P_115_-infected group. By 7 and 14 dpi, the majority of the mRNA exhibited similar down-regulation in both the P_5_-infected group and the P_115_-infected group. By 21 dpi, their change patterns were different: the mRNA level of these genes was up-regulated in the P_5_-infected group, but for the P_115_-infected group, they remained low at this time point. For genes from kidney tissue, as shown in Figure 5B, at 4 dpi, infection with the P_115 _strain resulted in a stronger up-regulation in the mRNA level than infection with the P_5 _strain. However, by 7 dpi, infection with P_5 _induced a stronger down-regulation than P_115 _infection. At 14 dpi, both virus strains resulted in a similar response, with the majority of mRNAs showing a down-regulation. In contrast, at 21 dpi, the majority of mRNAs were still down-regulated in the P_115_-infected group, but P_5 _infection induced up-regulation of the mRNA level of several genes.

In comparison with the results obtained using proteomics (summarized in Additional file 6 and Additional file 7), the trends in the mRNA levels of these genes were not completely consistent with the change patterns of their corresponding proteins in 2-DE or 2-DIGE gels. The disparity between the levels of mRNA and their corresponding proteins may occur because posttranscriptional and posttranslational modifications, as well as differential mRNA and protein degradation rates, may also contribute to these discrepancies. Several papers have addressed this question performing parallel proteomic/gene expression studies [23-26].

### Protein validation by western blot analysis

**Figure 6:**
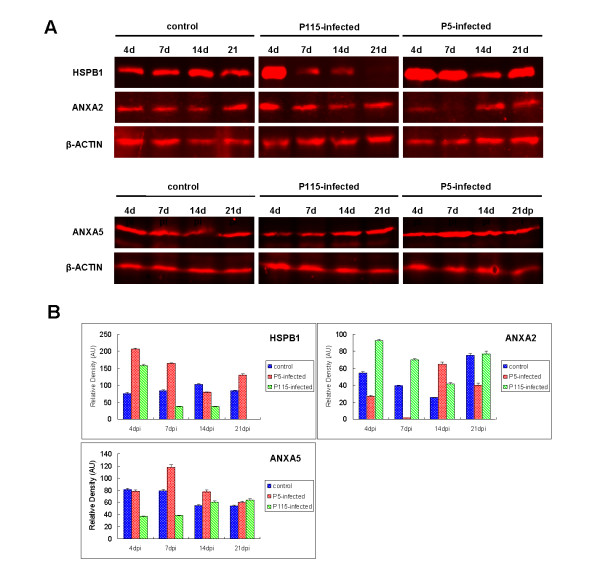
**(A) Western blot analysis of heat shock protein beta-1 (HSPB1) and annexin A2 (ANXA2) in trachea tissue, annexin A5 (ANXA5) in kidney tissue of chickens infected with the IBV ck/CH/LDL/97I P_5 _and ck/CH/LDL/97I P_115 _strain**. (B) Protein band density was analyzed with the BandScan software. Beta-Actin was used as the internal control. Mean values ± SE were calculated from three independent samples. AU, arbitrary units.

To confirm the dynamic alterations of protein abundance during infection with IBV ck/CH/LDL/97I P_5 _and ck/CH/LDL/97I P_115_, three proteins including annexin A2 (ANXA2), annexin A5 (ANXA5), and heat shock protein beta-1 (HSPB1) were selected for western blot analysis. As shown in Figure 6A and 6B, the abundance of HSPB1 was increased in trachea tissues from the P_5_-infected group at 4, 7, and 21 dpi, relative to the control group. It was also increased in the P_115_-infected group, relative to the control group. The abundance of Annexin A2 was increased in tracheal tissues from the P_115_-infected group at 4 and 7 dpi, but decreased in tracheal tissues from the P_5_-infected group at 7 dpi, relative to the control group. In addition, the abundance of annexin A5 was increased in P_5_-infected kidney tissues at 7 dpi, relative to the control group. These data were in agreement with the results obtained from the 2-DE and 2-DIGE analysis.

## Discussion

Viruses are obligatory intracellular pathogens that rely on the host cell for essentially all steps of their life cycle. Although coronaviruses use host proteins as part of their replication strategies, it has also become clear that the immune, metabolic, stress, cell cycling and other pathways are activated by infection. Determination, using genomics and proteomics, of the extent to which virus-host interaction is coronavirus-specific and organ-specific, will be of importance [14,27]. Our previous study on the global profile of host protein alterations in response to IBV infection was focused on an *in ovo *infection model system [21]. In this study, we applied a comparative proteomics technical platform for the first time to explore the abundance changed protein in trachea and kidney tissues from chicken infected *in vivo *with the highly virulent ck/CH/LDL/97I P_5 _strain of IBV and the embryo-passaged, attenuated ck/CH/LDL/97I P_115 _strain of IBV. Some identified proteins are likely to be important in the host response to virus infection, including cytoskeletal proteins, stress response proteins, and anti-oxidative proteins. Interestingly, the abundance of these proteins showed different change patterns with IBV strains differ in virulence, suggesting that some of these differences might be responsible for virulence, and consistent with our previous study which demonstrated the differences in pathology and virulence for these two different viruses [22,28]. These results provide an overview of the proteome profile of the host in response to different virulent IBV infection *in vivo*.

The cytoskeletal network is a cellular scaffold system whose functions include maintenance of cellular shape, enabling of cellular migration, cell division, intracellular transport, signaling, and membrane organization. Some host cytoskeletal proteins have been reported as differentially altered by virus infection in quantitative proteomic studies [16-18]. Recently, study has revealed that several different cellular proteins involved in cell morphology and the cytoskeleton changed in abundance in IBV infected cells [19,20]. IBV infection resulted in a number of changes to the nucleolus both in terms of gross morphology and protein content [29]. Our results presented a tendency that the abundance of several cytoskeletal proteins showed increased with the degree of virulence getting strong, which is possibly due to the collapse and dispersal of the cytoskeleton in the IBV-infected cells, as demonstrated by other viruses [30]. Vimentin is a major component of type III intermediate filaments that has been reported to be redistributed in cells around sites of virus replication and assembly during virus infection. For instance, infection with African swine fever virus (ASFV) can lead to the rearrangement of vimentin into a cage surrounding a virus factory, which may prevent movement of viral components into the cytoplasm and concentrate late structural proteins at sites of virus assembly [31]. Specifically, vimentin was observed to be increased in abundance in the cytoplasmic proteome of IBV-infected cells [19,20]. In our study, the abundance of vimentin was increased more strongly in the highly virulent P_5 _strain infected group, compared with the attenuated P_115 _strain infected group at 21 dpi. Given that vimentin can confer rigidity to domains of the cytoplasm, the vimentin cage may provide a physical scaffold to facilitate the construction of the virus factory. The stronger induction of vimentin could be a result of the large replication capacity of the highly virulent IBV strain. Tropomyosin belongs to the family of actin-binding proteins that serve important functions in microfilament stabilization, regulation of microfilament branching, actin polymerization, and intracellular transport. The abundance of alpha-tropomyosin was found increased in IBV-infected cell [19,20]. In our study, the abundance of alpha-tropomyosin was increased much more strongly with highly virulent IBV ck/CH/LDL/97I P_5 _infection than with the attenuated P_115 _strain, it is likely that it help to orchestrate virus assembly, release and efficient cell-to-cell spread, also may due to the different virulence between IBV ck/CH/LDL/97I P_5 _and P_115 _strain. In addition, in our previous *in ovo *infection model [21], the abundance of some cytoskeletal including alpha-tropomyosin was decreased in the IBV-infected embryonic tissues, this difference need to be further investigated.

In both our previous [21] and current study, the abundance of annexin A2 and annexin A5 were found to be changed upon IBV infection. Annexins are a family of conserved proteins characterized by their ability to bind and order charged phospholipids in membranes, often in response to elevated intracellular calcium. These family members are involved specifically in a diverse range of cellular functions both inside the cell and extracellularly [32]. Annexin A2 (ANXA2) can associate with actin filaments and mediates membrane trafficking and membrane-cytoskeletal interactions. It has been identified as an important host factor for several viruses and at different stages of their life cycle. ANXA2 is incorporated into cytomegalovirus and influenza virus particles, promotes the entry of virus, and plays a role in Human immunodeficiency virus -1 (HIV-1) assembly, Bluetongue virus (BTV) release, and hepatitis C virus (HCV) replication [33-40]. Annexin A5 (ANXA5) is involved in various intra- and extracellular processes including signal transduction, anti-inflammatory processes, membrane trafficking, and ion channel activity [41], it also acts to regulate blood coagulation, binding to and shielding exposed phospholipids and masking their pro-thrombotic properties [42]. The expression of ANXA2 and ANXA5 has been found to be altered during other IBV infection system [19,20]. In this study, the abundance of ANXA2 showed weaker increase at early stages of infection with the highly virulent IBV ck/CH/LDL/97I P_5 _strain than with the attenuated P_115 _strain. For ANXA5, the level of expression induced at 7 dpi with the highly virulent P_5 _strain of IBV was greater than that induced by the attenuated P_115 _strain. These trends were also validated by western blot analysis. Whether this difference between P_5 _and P_115 _infectioned groups is related with the virulence of IBV or various responses of the host cell deserves further study.

MnSOD is the primary antioxidant enzyme in the mitochondria that catalyzes the conversion of superoxide molecules to hydrogen peroxide and molecular oxygen and therefore forms one of the cell's major defense mechanisms against oxidative stress [43]. Studies have reported that viral infections cause oxidative stress, which is associated with the activation of phagocytes and an increase in the release of reactive oxygen species (ROS) that play a positive modulatory role in immune activation, the inflammatory response, eradication of viral infections and immunity-induced cellular injury [44]. The expression of MnSOD was altered during infection with some viruses, such as porcine reproductive and respiratory syndrome virus, influenza A virus, and SARS-CoV [45-47]. The abundance of the anti-oxidative protein MnSOD was increased much significantly in highly virulent IBV ck/CH/LDL/97I P_5 _infected group than the attenuated P_115 _strain infected group. The much amplitude increased production of MnSOD during highly virulent strain infection can affect the host cell pro-/anti-oxidant balance, which probably results in more significant immune activation and a stress response induced by reactive oxygen species. It is important in the eradication of viral infections and immune-induced cellular injury.

When comparing the protein profiles of groups infected with different strains, we found that the abundance of HSPB1, a well-known heat-shock protein, in highly virulent IBV ck/CH/LDL/97I P_5 _strain infected group was significantly higher than the embryo-passaged, attenuated P_115 _strain at the early stage of infection. Western blot and real-time RT-PCR further confirmed this alteration. HSPB1 has different cytoprotective roles, including acting as a molecular chaperone, maintaining the normal function of cells through interaction with and stabilization of the cytoskeleton, regulation of translational initiation, modulation of inflammation, inhibition of apoptosis, stimulation of innate and adaptive immune responses, and responding to a wide variety of stressful stimuli [48-50]. It is probably involved in all phases of the viral life cycle, including cell entry, virion disassembly, viral genome transcription, replication and morphogenesis. The abundance of HSPB1 was observed to be increased in IBV-infected Vero cells using stable isotope labeling with amino acids in cell culture (SILAC) [20]. IBV infection induces cell cycle arrest at both S and G2/M phases and caspase-dependent apoptosis at late stages of the viral infection cycle [51-53]. Results presented in our study were probably due to the highly virulent virus triggering a markedly more robust inflammation and stress response. It also suggests that many important, and probably different events in IBV pathogenesis and immunology, such as the stress response, inflammation, and apoptosis, occur early in infection, and that these events may contribute to development of an appropriate immune response and the outcome of viral infection. In our previous study [21], the abundance of HSPB1 was found to be decreased after IBV infection *in ovo*. This difference in different infection model need to be further examined.

In this study, infection with both the highly virulent strain and the embryo-passaged, attenuated strain of IBV was accompanied by elevation of proteins related to energy metabolism. Viruses are obligate parasites that are completely dependent on their host's cellular metabolism for reproduction. Viral infection has been shown to modulate the levels of numerous host metabolic components in pathways such as glycolysis, the tricarboxylic acid (TCA) cycle, pentose phosphate pathway, and macromolecular biosynthesis in order to redirect valuable resources to their own mass production. Studies have demonstrated that the rate of glycolysis in cells infected by Rous sarcoma virus, feline leukemia virus, and poliomyelitis virus was increased by as much as 370% [54]. Glycolytic enzymes are involved not only in carbohydrate metabolism and increased ATP production, also take part in the induction of anti-oxidative stress in host cells and contribute to transcription of RNA virus genomes [55]. The abundance of some proteins involved in glycolysis, such as aldose reductase, pyruvate kinase, alpha-enolase, and triosephosphate isomerase was found increased in IBV infected cell [19,20]. Our study also showed that the abundance of some glycolytic enzymes, including phosphoenolpyruvate carboxykinase (PCK1), mitochondrial phosphoenolpyruvate carboxykinase (PCK2), pyruvate kinase, and alpha-enolase, were increased significantly in the early stage of infection with both the highly virulent and the attenuated IBV strain. These findings support the view that IBV infection probably triggers activation of host energy metabolic components via systemic or global mechanisms, to keep up with the energy demands of its own replication.

The post-translational modification (PTM) plays critical roles in cellular regulation. It has been reported that PTM and expression of highly related gene sequences can induce multiple protein spots in animals [56]. An advantage of 2-DE is the monitoring of multiple forms of a protein species, which offers the opportunity to investigate the effects of the virus infection on protein modification. Our study revealed that several proteins were expressed by multiple spots in our study. Although we do not know at present how these multiple spots were generated, some of them were more likely due to post-translational modifications (PTMs), or highly related gene sequences coding isoforms [57].

## Conclusion

In summary, we investigated the proteome profiles of tracheal and kidney tissues from chicken infected with highly virulent IBV ck/CH/LDL/97I P_5 _and embryo-passaged, attenuated IBV ck/CH/LDL/97I P_115_. Some proteins involved in cytoskeleton organization, stress response, and anti-oxidative stress, showed different change in abundance with IBV strains differing in virulence. While the roles of some identified alterations could be related to host antiviral response or pathogenic mechanisms, functional significance of other altered proteins remains unclear and needs further investigation. However, our findings provide proteome-related information on a large scale that should be useful in increasing our understanding of the pathogenic and immune mechanisms of IBV infection. In addition, they will also provide reference for similar research into other coronaviruses.

## Materials and methods

### Experimental animals and infecting virus strains

One-day-old White Leghorn specific-pathogen-free (SPF) chickens were obtained from the Laboratory Animal Center, Harbin Veterinary Research Institute, the Chinese Academy of Agricultural Sciences, China. The chickens were maintained in isolators with negative pressure, and food and water were provided *ad libitum*. This study was approved by the Animal Welfare Committee of Heilongjiang Province, China.

The highly virulent ck/CH/LDL/97I P_5 _strain of IBV and attenuated IBV strain ck/CH/LDL/97I P_115 _were obtained by passaging the IBV ck/CH/LDL/97I strain, which was isolated and identified by our laboratory as described previously [22]. The IBV strains were propagated once in 9- to 11-day-old embryonated SPF chicken eggs for preparation of seed stock. The presence of viral particles in the allantoic fluid of inoculated eggs was confirmed with a negative contrast electronic microscope (JEM-1200, EX) as described previously [22].

### Experimental design

Sixty-six one-day-old SPF White Leghorn chickens were divided randomly into three groups: the P_5_-infected group, P_115_-infected group and control group. Twenty-two chickens were allocated to each group and housed in different isolators. Chickens in the P_5_-infected and P_115_-infected groups were inoculated with the IBV ck/CH/LDL/97I P_5 _strain and ck/CH/LDL/97I P_115 _strain, respectively, by oculo-nasal application at 11 days of age with a dose of log10^6 ^EID_50 _per chicken. The chickens in the control group were mock-inoculated in parallel with sterile allantoic fluid.

Blood samples from 10 chickens in each group were collected at 4, 7, 14, and 21 days post inoculation. The sera were collected for enzyme-linked immunosorbent assay (ELISA) testing. Three chickens were selected randomly from each group and killed humanely at 4, 7, 14, and 21 days post inoculation, respectively. Trachea and kidney tissues were separated rapidly and washed with ice-cold PBS buffer, snap-frozen in liquid nitrogen, and kept subsequently at -80°C for 2-DE or 2-DIGE, real-time RT-PCR, and western blot analysis.

### Serum antibody detection

Serum samples were assayed using a commercial IBV antibody test kit (IDEXX Corporation, Westbrook, Maine, USA) according to the manufacturer's instructions. Each sample was tested in triplicate. Serum-to-positive ratios (S/P ratios) were calculated as described previously [2,58]. Individual serum titers were calculated from these S/P ratios, evaluated as positive or negative, and expressed as OD_650 nm _values according to the manufacturer's instructions.

### Quantitative analysis of IBV in trachea and kidney by real-time RT-PCR

Viral load of IBV was analyzed using R. M. Jones described methods [59]. Tissue samples were ground and homogenized, 100 mg tissue homogenates were suspended in 500 μl phosphate-buffered saline (PBS) containing 100 μg penicillin and 100 μg streptomycin/ml, the suspension were freezed thawing three times, then centrifuged at 13,000 × g at 4°C for 5 minutes. RNA was extracted from 200 μl tissue supernatant using TRIzol Reagent (Invitrogen) following the manufacturer's instructions. The real-time RT-PCR assay used the following primer and probe sequences: IBVF forward primer CTA TCG CCA GGG AAA TGT C, IBVR reverse primer GCG TCC TAG TGC TGT ACC C, IBV TaqMan^® ^probe FAM-CCTGGAAACGAACGGTAGACCCT-TAMRA [59]. One-step real-time RT-PCR reactions were performed using One Step PrimeScript^® ^RT-PCR kit (TaKaRa Biotech Co. Ltd., Dalian) on LightCycler^® ^480 real-time PCR system (Roche) according to the following steps: reverse transcription at 42°C for 10 min, denaturation at 95°C for 10 s. and 40 cycles with 95°C for 5 s, 55°C for 20 s, 72°C for 10 s, followed by a 40°C for 10 s cooling step. All of the samples were tested in triplicate in each reaction. The data were analyzed using LightCycler^® ^480 Software Version 1.5.

### Preparation of protein samples

The frozen trachea or kidney tissues were placed in liquid nitrogen and ground thoroughly to a very fine powder. Samples of 100 mg tissue powder were dissolved in 500 μl lysing solution containing 7 M urea, 2 M thiourea, 4% CHAPS, 40 mM DTT, 2% IPG buffer pH 3-10, 1% Nuclease Mix and 1% Protease Inhibitor Mix (GE Healthcare), then incubated for 2 h at 4°C with vortexing once every 15 min, and centrifuged at 15,000 × g for 1 h at 4°C. The supernatant was collected and purified with the PlusOne 2D Clean-up kit (GE Healthcare). The concentration of each protein sample was determined with the PlusOne 2D Quant Kit (GE Healthcare). Protein samples were aliquoted and stored at -80°C for 2-DE or 2-DIGE analysis.

### 2-DE analysis of trachea protein samples

Thirty-six samples of tracheal protein from the three groups (P_5_-infected, P_115_-infected and control) at 4, 7, 14, and 21 days post-inoculation (dpi) were analyzed by 2-DE using the methods previously described [21]. First, 400 μg protein samples were added to rehydration solution (7 M urea, 2 M thiourea, 40 mM DTT, 2% CHAPS, 0.5% pH 3-10 or pH 4-7 IPG buffer, and 0.002% bromophenol blue) to make the final volume up to 250 μl, following Isoelectric focusing (IEF), the IPG strips were equilibrated and the second dimension separation was performed on 12.5% SDS-polyacrylamide gels on the SE600 Ruby system (GE Healthcare). Then, the gels were stained with PlusOne Coomassie Blue R-350 (GE Healthcare) and scanned with an ImageScanner III (GE Healthcare). Quantification analyses were performed with Image Master 2D Platinum software v6.0 (GE Healthcare). For image analysis, three independent gels from the P_5_-infected and P_115_-infected groups were compared with those from the corresponding control group at 4, 7, 14, and 21 dpi respectively. The normalized volume values (vol %) of matched protein spots were subjected to Student's *t *test using the SPSS statistical software package version 16.0. The criterion used to define differential expression of spots was that the ratio of the vol % in the P_5_-infected group or the P_115_-infected group vs. the control group was more than 1.5 (p < 0.05) or less than 0.67 (p < 0.05). Differentially changed protein spots were excised manually from the gels and subjected to MS analysis.

### 2-DIGE analysis of kidney protein samples

The kidney protein samples from each group at 4, 7, 14, and 21 dpi were used for 2-DIGE analysis respectively. The pH of the protein samples was adjusted to 8.5 and the protein concentration was adjusted to 5 μg/μl. Equal amounts of protein from each sample were pooled together as the internal standard. The proteins were minimally labeled according to the manufacturer's instructions (CyDye DIGE fluor minimal labeling kit, GE Healthcare). The Cy2 was used to label the pooled internal standard, and Cy3 and Cy5 were used randomly to label samples from the control group, P_5_-infected group and P_115_-infected group. To minimize system error and inherent biological variation, the sample multiplexing was also randomized (see Additional file 8) to produce unbiased results. Following the labeling reaction, 50 μg of each Cy2, Cy3, and Cy5 labeled sample was mixed, the pooled sample of each gel was adjusted to 450 μl with rehydration buffer (7 M urea, 2 M thiourea, 2%CHAPS, 2%v/v IPG buffer,130 mM DTT), and loaded subsequently onto 24 cm, linear pH 3-10 IPG strips (GE Healthcare). Isoelectric focusing (IEF) was performed on an Ettan™ IPGphor 3 (GE Healthcare) using the following protocol: 30 V for 12 h, 200 V for 2 h, 500 V for 2 h, 1000 V for 2 h, 8000 V for 3 h, and 8000 V for 65000 Vh. Then, the IPG strips were equilibrated and the second dimension separation was conducted on 12.5% SDS-polyacrylamide gels using an Ettan™ DALT six system (GE Healthcare). The CyDye-labeled gels were scanned using a Typhoon 9400 (GE Healthcare). Image analysis was performed using Ettan™ DeCyder Software version v6.5 (GE Healthcare) as described in the user's manual. The statistical analysis of changes in protein abundance in different gels was performed on the basis of the spot volumes. Protein spots with statistically significant results for the Student's *t*-test (p < 0.05) and Average Ratio more than1.5 or less than 0.67 were considered to be differentially changed protein spots.

The preparative gels were made with 1200 μg of unlabeled proteins (400 μg from each group). These mixed proteins were loaded and separated under the same conditions as described above. The preparative gels were stained with Coomassie Blue R-350. Each spot of interest, defined on the basis of CyDye images, was matched with a Coomassie Blue R-350 image, then excised manually from the gel and subjected to MS analysis.

### MALDI-TOF/TOF MS and database search

The gel samples were placed in a tube and washed twice with 500 μl and 250 μl ddH_2_O for 15 min. For trypsin digestion, the gel samples were washed twice with 50 mM w/v NH_4_HCO_3 _and covered with 10 mg/ml Porcine Trypsin solution (Promega, Madison, WI, USA) in 50 mM w/v NH_4_HCO_3_. After incubation overnight at 37°C, the supernatant was removed into a second tube and 40 μl 50 mM w/v NH_4_HCO_3 _was added. Gel samples were washed with 40 μl of 50 mM w/v NH_4_HCO_3_, the supernatant was collected, and both collected supernatants were combined. The gel was washed with 70% v/v ACN and dried in a Speed Vac (Vacuum Concentrator, Bachhofer). The peptide mixtures were desalted using ZipTip C-18 RP tips (Millipore, Billerica, MA, USA) which were wetted with 100% ACN and equilibrated with 0.1% TFA. Peptide samples, which were redissolved in 10 ml 0.5% TFA, were eluted with 50% ACN/0.1% TFA and dried in a Speed Vac (Vacuum Concentrator).

The purified peptides were spotted on a MALDI plate and covered with 0.7 μl of 2 mg/ml 3, 5-Dimethoxy-4-hydroxycinnamic acid matrix (Sigma) with 10 mM NH_4_H_2_PO_4 _in 60% ACN. All samples were analyzed by MALDI-TOF/TOF MS with a 4700 Proteomics Analyzer (Applied Biosystems, Foster City, CA). Monoisotopic peak masses were acquired in a mass range of 800-4000 Da, with a signal-to-noise ratio (S/N) of 200. Five of the most intense ion signals, excluding common trypsin autolysis peaks and matrix ion signals, were selected as precursors for MS/MS acquisition. The peptide mass fingerprint (PMF) combined MS/MS data were submitted to MASCOT version 3.0 (Matrix Science) for identification according to the NCBInr database (release 16/01/2010, 10343571 sequences, 3528215794 residues). The search parameters were set as follows: Gallus, trypsin cleavage (one missed cleavage allowed), carbamidomethylation of cysteine as fixed modification, oxidation of methionine as variable modification, peptide mass tolerance set at 100 ppm, fragment tolerance set at 0.8 Da. The criterion for successful identification of a protein was the protein score confidence interval (C.I. %) ≥ 95%.

### Gene ontology (GO) annotation of differentially expressed proteins

Spot identities were submitted to *GORetriever *http://www.agbase.msstate.edu/ to obtain the GO annotations. If no annotation was returned, *GOanna *was used to retrieve GO annotations assigned on the basis of sequence similarities. The resulting annotations were summarized according to the GOA and whole proteome GOSlim set using *GOSlimViewer *[60].

### Analysis of mRNA levels by real-time RT-PCR

Specific primers were designed according to the corresponding gene sequences of the MS-identified proteins using Beacon Designer software 7.5 (Primer Biosoft International). Information on the primers is listed in Additional file 9. Total RNA was extracted using TRIzol Reagent (Invitrogen) according to the manufacturer's instructions. Two micrograms of total RNA was reverse transcribed with 200 U M-MLV Reverse Transcriptase (Invitrogen) and 500 ng Oligo(dT)_18 _as the first strand primer in 20 μl reaction solution. Real-time RT-PCR was carried out using SYBR^® ^Premix Ex Taq™ II kit (Takara) on the LightCycler^® ^480 real-time PCR system (Roche) according to the following steps: 30 s at 95°C, 40 cycles of denaturation at 95°C for 10 s, and annealing and extension at 55°C for 45 s. Each sample was amplified in triplicate. Quantitative analysis of the data was performed using the 2^-ΔΔCt ^method [61]; samples from the control group at 4 dpi were used as the calibrator (relative expression = 1), and the 18S ribosomal RNA gene was used as an internal reference gene.

### Western blot analysis

Samples of tracheal and kidney proteins from the P_5_-infected group, P_115_-infected group, and control group at all four time points were prepared, and the protein concentration was determined as described above. Equivalent amounts of total protein were subjected to 12% SDS-PAGE and then transferred to a nitrocellulose membrane. After blocking for 1 h at 37°C, the membranes were incubated separately with mouse monoclonal antibody to annexin A5 (sc-32321, Santa Cruz Biotechnology, USA), mouse monoclonal antibody to HSPB1 (sc-51956, Santa Cruz Biotechnology, USA), and goat polyclonal antibody to annexin A2 (sc-1924, Santa Cruz Biotechnology, USA) overnight at 4°C. The membranes were incubated subsequently with IRDye700DX conjugated anti-mouse secondary antibody (610-130-121, Rockland, Gilbertsville, PA) for 1 h at 37°C, and scanned finally on a LI-COR infrared imaging system using their Odyssey software (Li-Cor Bioscience, Lincoln, NE). β-actin (sc-47778, Santa Cruz Biotechnology, USA) was used as a reference protein to check equal loading. Triplicate experiments were performed for each sample. Densitometric analysis of protein bands was done by using BandScan software (Glyko).

## Abbreviations

DPI: Days post infection; ACN: Acetonitrile; CHAPS: 3-[(3-cholamidopropyl) dimethyl-ammonio] -1-propanesulfonate; DTT: Dithiothreitol; IBV: Infectious bronchitis coronavirus; IEF: Isoelectric focusing; IPG: Immobilized pH gradient; MALDI-TOF-TOF/MS: Matrix-assisted laser desorption/ionization time-of-flight tandem mass spectrometry; PMF: Peptide mass fingerprinting; SDS-PAGE: Sodium dodecyl sulfate polyacrylamide gel electrophoresis; SPF: Specific pathogen free; TFA: Trifluoroacetic acid; 2-DE: Two-dimensional gel electrophoresis; 2-DIGE: Two-dimensional fluorescence difference gel electrophoresis.

## Competing interests

The authors declare that they have no competing interests.

## Authors' contributions

SL designed the study. SL and ZC drafted the manuscript. ZC, ZH, YS, and XL carried out virus infection, serum antibody detection, and quantitative analysis of IBV. ZC, JS and DY carried out the 2-DE experiments, image analysis, excised the protein spots, data analysis and interpretation, confirmed the differential expression by real-time RT-PCR and Western blotting analysis. SL wrote the manuscript. XK revised the manuscript. All authors read and approved the final manuscript.

## Supplementary Material

Additional file 1 **Table S1 Serological results post inoculation with IBV ck/CH/LDL/97I P_5 _and ck/CH/LDL/97I P_115_**. Click here for file

Additional file 2 **Figure S1 Summary of changes in protein levels over time following infection with IBV ck/CH/LDL/97I P_5 _and P_115_**. The y axis shows the number of differentially expressed protein spots; individual spots can be found in Tables 1, 2, 3, and 4. Click here for file

Additional file 3 **This includes the PMF spectrum and confirmed MALDI-TOF-TOF spectrum of differentially expressed protein spots in IBV-infected chick tracheal tissues**. Click here for file

Additional file 4 **This includes the PMF spectrum and confirmed MALDI-TOF-TOF spectrum of differentially expressed protein spots in IBV-infected chick kidney tissues**. Click here for file

Additional file 5 **Table S2 Biological function of differentially expressed proteins reported in viral infection**. Click here for file

Additional file 6 **Table S3 Comparison of the fold changes for protein abundance observed by 2-DE gel analysis and mRNA level obtained by real-time RT-PCR in trachea tissues**. Click here for file

Additional file 7 **Table S4 Comparison of the fold changes for protein abundance observed by 2-DIGE gel analysis and mRNA expression obtained by real-time RT-PCR in kidney tissues**. Click here for file

Additional file 8 **Table S5 Experiment design of the different fluorescent dye labeling**. Click here for file

Additional file 9 **Table S6 The primers of Real-time RT-PCR**. Click here for file
