# Supplementary material for: Proteomics analysis of differentially expressed proteins in chicken trachea and kidney after infection with the highly virulent and attenuated coronavirus infectious bronchitis virus in vivo
Source: Proteome Sci. 2012 Mar 31;10:24. doi: 10.1186/1477-5956-10-24 (PMC3342233; doi:10.1186/1477-5956-10-24)
Supplement: Additional file 1 — Table S1 Serological results post inoculation with IBV ck/CH/LDL/97I P5 and ck/CH/LDL/97I P115. [file 1477-5956-10-24-S1.DOC]

Table S1 Serological results post inoculation with IBV ck/CH/LDL/97I P_5_ and ck/CH/LDL/97I P_115_

| Group^a^ | Inoculated | Dose, median embryo infectious doses (log 10)^b^ | Morbidity | Mortality | Antibody^c^ | | | |
| --- | --- | --- | --- | --- | --- | --- | --- | --- |
|  |  |  |  |  | Days post inoculation | | | |
|  |  |  |  |  | 4 | 7 | 14 | 21 |
| Control | No | - | 0/10 | 0/10 | 0/10 | 0/10 | 0/10 | 0/10 |
| P_5_ | Yes, with CK/CH/LDL97I P_5_ | 6.0 | 10/10 | 0/10 | 0/10 | 3/10 | 10/10 | 10/10 |
| P_115_ | Yes, with CK/CH/LDL97I P_115_ | 6.0 | 0/10 | 0/10 | 0/10 | 1/10 | 10/10 | 10/10 |

a) Twenty-two chickens per group.

b) Dose per chick (ck/CH/LDL/97I P_5_ and ck/CH/LDL/97I P_115_), 100 μl.

c) Number seroconverted/number inoculated.
