# Supplementary figures and images for: Proteomics analysis of differentially expressed proteins in chicken trachea and kidney after infection with the highly virulent and attenuated coronavirus infectious bronchitis virus in vivo
Source: Proteome Sci. 2012 Mar 31;10:24. doi: 10.1186/1477-5956-10-24 (PMC3342233; doi:10.1186/1477-5956-10-24)

## Slide 1
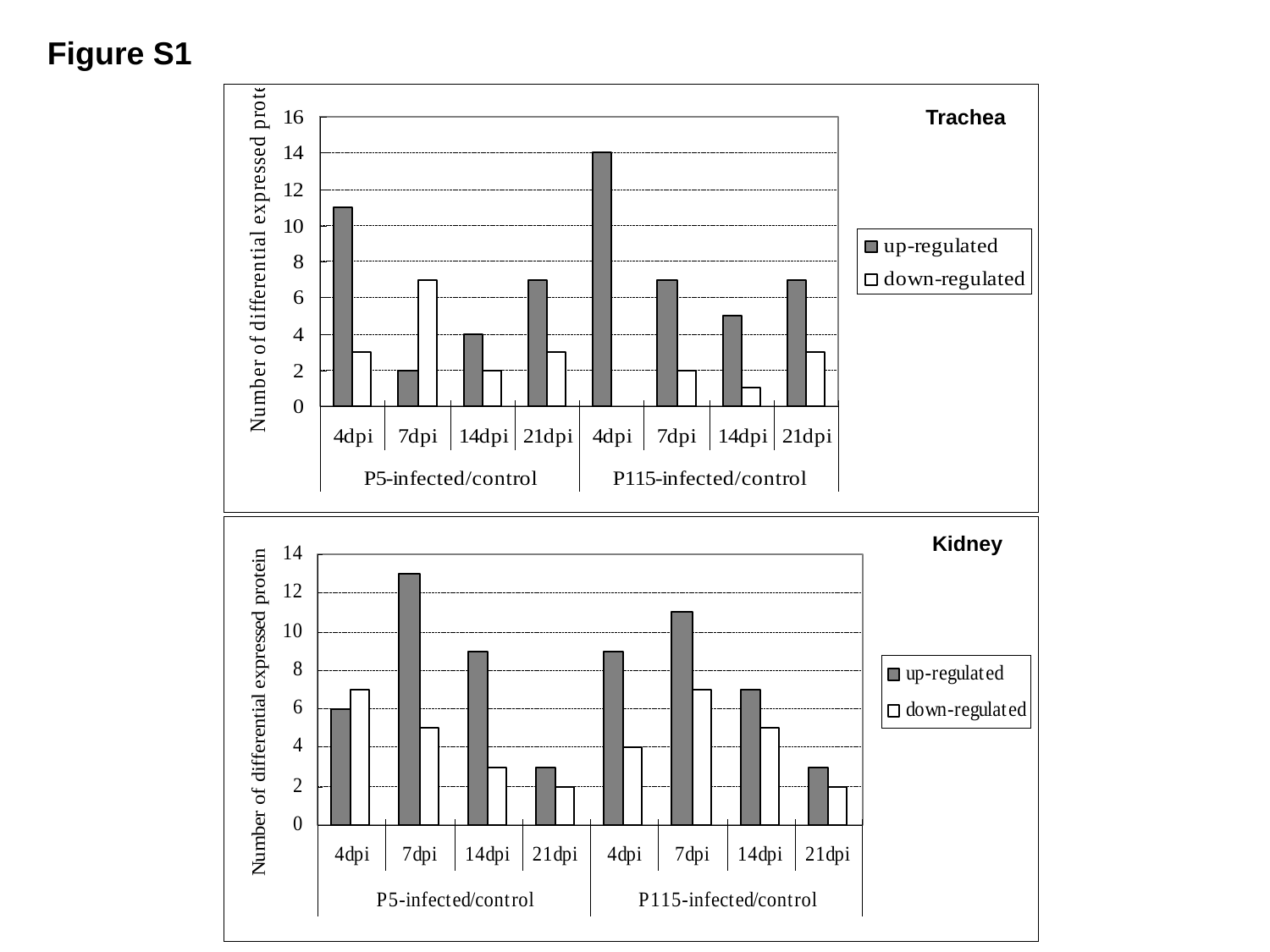

Figure S1
Trachea
Kidney

Supplement: Additional file 2 — Figure S1 Summary of changes in protein levels over time following infection with IBV ck/CH/LDL/97I P5 and P115. The y axis shows the number of differentially expressed protein spots; individual spots can be found in Tables 1, 2, 3, and 4. [file 1477-5956-10-24-S2.PPT]
