# Supplementary material for: Proteomics analysis of differentially expressed proteins in chicken trachea and kidney after infection with the highly virulent and attenuated coronavirus infectious bronchitis virus in vivo
Source: Proteome Sci. 2012 Mar 31;10:24. doi: 10.1186/1477-5956-10-24 (PMC3342233; doi:10.1186/1477-5956-10-24)
Supplement: Additional file 5 — Table S2 Biological function of differentially expressed proteins reported in viral infection. [file 1477-5956-10-24-S5.DOC]

**Table S2 Biological function of differentially expressed proteins reported in viral infection**

| Protein description | Biological function | Reported in other virus infections^*^ |
| --- | --- | --- |
| PREDICTED: similar to cystathionase [*Gallus gallus*] | amino acid metabolism |  |
| PREDICTED: similar to CDNA sequence BC048390 [*Gallus gallus*] | catalytic activity |  |
| PREDICTED: similar to guanidinoacetate N-methyltransferase [*Gallus gallus*] | amino acid metabolism |  |
| manganese-containing superoxide dismutase precursor [*Gallus gallus*] | anti-oxidative stress | RSV, MV, IAV, SARS-CoV, EBV, PRRSV |
| chain A, the structure of chicken mitochondrial Pepck in complex with Pep | carbohydrate metabolism | HBV, SARS-CoV |
| B-creatine kinase [*Gallus gallus*] | amino acid metabolism |  |
| phosphoenolpyruvate carboxykinase (EC 4.1.1.32) [*Gallus gallus*] | carbohydrate metabolism |  |
| triosephosphate isomerase [*Gallus gallus*] | carbohydrate metabolism | IAV, WSSV, DENV, IBDV |
| alpha-enolase [*Gallus gallus*] | carbohydrate metabolism | DENV, SARS-CoV, HIV, IBDV, PRRSV, PCV2, WSSV, HSV-1, RSV, DHBV, HBV, VHSV, WNV, |
| similar to methyltransferase 24 | RNA processing and biosynthesis | IBDV |
| catalase [*Gallus gallus*] | anti-oxidative stress | DENV |
| ovotransferrin BB type [*Gallus gallus*] | iron ion transport |  |
| aldehyde dehydrogenase 4 family, member A1 | oxidoreductase activity |  |
| PREDICTED: similar to aflatoxin aldehyde reductase[*Gallus gallus*] | oxidoreductase activity |  |
| glutamate dehydrogenase 1,mitochondrial | amino acid metabolism |  |
| chain A, transthyretin | transmembrane transporter activity |  |
| fibrinogen γ chain | acute phase response, blood coagulation, signal transduction | CVB3, SARS-CoV, HBV |
| nucleoside diphosphate kinase [*Gallus gallus*] | nucleotide biosynthesis |  |
| retinal dehydrogenase 1 [*Gallus gallus*] | oxidoreductase activity |  |
| sulfotransferase [*Gallus gallus*] | transferase activity |  |
| PREDICTED: similar to betaine homocysteine methyltransferase [*Gallus gallus*] | amino acid biosynthesis |  |
| annexin A5 | calcium ion binding, signal transduction | PRRSV, DV, DHBV, HBV, VHSV, IAV, HIV, HCMV |
| cytochrome c oxidase subunit 4 isoform 1,mitochondrial [*Gallus gallus*] | oxidoreductase activity, mitochondrial respiratory chain |  |
| alpha-tropomyosin of smooth muscle [*Gallus gallus*] | motor activity, structural constituent of cytoskeleton, actin binding and stabilization | HPV8, PRRSV, RSV, HPV8, VHSV, CVB3 |
| low molecular weight phosphotyrosine proteinphosphatase [*Gallus gallus*] | protein metabolism, signal transduction | HCV |
| PREDICTED: similar to dodecenoyl-coenzyme A delta isomerase [*Gallus gallus*] | fatty acid beta-oxidation |  |
| D-amino-acid oxidase | oxidoreductase activity |  |
| receptor-associated protein [*Gallus gallus*] | lipid metabolism |  |
| class I alcohol dehydrogenase, beta subunit [*Gallus gallus*] | carbohydrate metabolism |  |
| carbonyl reductase [NADPH] 1 [*Gallus gallus*] | lipid metabolism |  |
| apolipoprotein A-I | lipid metabolism | HIV, CVB3, SARS-CoV, IBDV |
| aldehyde dehydrogenase 2 family (mitochondrial) [*Gallus gallus*] | oxidation reduction | PRRSV |
| aldo-keto reductase [*Gallus gallus*] | oxidoreductase activity | IBDV |
| fibrinogen β chain [*Gallus gallus*] | acute phase response, blood coagulation, signal transduction | CVB3, SARS-CoV, HEV, HCV |
| cartilage matrix protein precursor [*Gallus gallus*] | extracellular matrix structural constituent |  |
| pyruvate kinase [*Gallus gallus*] | carbohydrate metabolism, glycolysis | IAV, SARS-CoV, HIV, HCV, RSV |
| annexin A2 [*Gallus gallus*] | calcium ion binding, signal transduction, virus entry; egress and secretion | HBV, WNV, PRRSV, MHV, CSFV, HIV, DHBV |
| heat shock protein beta-1 [*Gallus gallus*] | chaperone, apoptosis, stress response | PrV, EBV, MV, HIV, IAV, IBDV, CSFV, PRRSV, PCV2, ASFV, AIV, MDV, REOV, CVB3 |
| succinyl-CoA:3-ketoacid-coenzyme A transferase 1,mitochondrial [*Gallus gallus*] | carbohydrate metabolism |  |
| nmrA-like family domain-containing protein 1 [*Gallus gallus*] | oxidation reduction |  |
| creatine kinase M-type [*Gallus gallus*] | amino acid metabolism | HBV, VHSV |
| keratin, type I cytoskeletal 19 [*Gallus gallus*] | intermediate filament cytoskeleton | RSV |
| PREDICTED: similar to malate dehydrogenase 2,NAD (mitochondrial) [*Gallus gallus*] | carbohydrate metabolism |  |
| UMP-CMP kinase [*Gallus gallus*] | nucleic acid metabolism |  |
| fructose-bisphosphate aldolase C | carbohydrate metabolism, glycolysis | WSSV, DENV |
| PREDICTED: microfibril-associated glycoprotein 4 (EST) | extracellular matrix protein |  |
| B-creatine kinase [*Gallus gallus*] | amino acid metabolism |  |
| vimentin [*Gallus gallus*] | intermediate filament cytoskeleton, virus trafficking | HSV-1, RV, WNV, PRRSV, IAV, IBDV, SARS-CoV, IBV, DV2, BTV, HIV, EBV, HTLV-I, HCV, HBV |
| PREDICTED: similar to D4-GDP-dissociation inhibitor | signal transduction | IBDV, CSFV, RSV, WSSV, YHV |
| lamin-A [*Gallus gallus*] | intermediate filament cytoskeleton, Interacts with actin in the nucleus | RSV, HSV-1, PrV, PRRSV, HPV8, MHV, CVB3, DHBV, HCMV |
| annexin A1 [*Gallus gallus*] | calcium ion binding, signal transduction, vesicle transport | HBV, WNV, PRRSV, IAV, CSFV, PRRSV, RSV, VHSV |
| glutathione S-transferase class-alpha [*Gallus gallus*] | detoxifying processes |  |
| PREDICTED: similar to myozenin [*Gallus gallus*] | calcineurin-binding protein |  |

*) IAV, Influenza A virus; RSV, Human respiratory syncytial virus; CVB3, coxsackievirus B3; VHSV, Viral hemorrhagic septicemia virus; CSFV, classical swine fever virus; PRRSV, Porcine reproductive and respiratory syndrome virus; PCV2, Porcine circovirus type 2; ASFV, African swine fever virus; HBV, Hepatitis B virus; AIV, Avian influenza virus; EV71, Enterovirus 71; MDV, Marek’s disease virus; WSSV, White spot syndrome virus; DHBV, Duck hepatitis B virus; YHV, yellow head virus; HPV8, Human papillomavirus type 8; REOV, Reovirus; IBDV, Infectious bursal disease virus; HCV, Hepatitis C virus; VSV, Vesicular stomatitis virus; HSV-1, Herpes simplex virus type-1; DV, Dengue virus; HIV, Human immunodeficiency virus; SARS-CoV, Severe acute respiratory syndrome-associated coronavirus; VHSV, Viral hemorrhagic septicemia virus; WNV, West Nile virus; BVDV, Bovine viral diarrhea virus; EBV, Epstein–Barr virus; MV, mumps virus; PrV, Pseudorabies virus; RV, rabies virus; HCMV, Human cytomegalovirus; HEV, Hepatitis E virus; DV2, Dengue virus 2; BTV, Bluetongue virus; HTLV-I, Human T-cell leukemia virus type I.
