# Supplementary material for: Proteomics analysis of differentially expressed proteins in chicken trachea and kidney after infection with the highly virulent and attenuated coronavirus infectious bronchitis virus in vivo
Source: Proteome Sci. 2012 Mar 31;10:24. doi: 10.1186/1477-5956-10-24 (PMC3342233; doi:10.1186/1477-5956-10-24)
Supplement: Additional file 6 — Table S3 Comparison of the fold changes for protein abundance observed by 2-DE gel analysis and mRNA level obtained by real-time RT-PCR in trachea tissues. [file 1477-5956-10-24-S6.DOC]

Table S3 Comparison of the fold changes for protein abundance observed by 2-DE gel analysis and mRNA level obtained by real-time RT-PCR in trachea tissues

| dpi | Gene symbol | P5-infected/control | | | | P115-infected/control | | | |
| --- | --- | --- | --- | --- | --- | --- | --- | --- | --- |
|  |  | 2-DE | | Real-time RT-PCR | | 2-DE | | Real-time RT-PCR | |
|  |  | ratio | *p* | ratio | *p* | ratio | p | ratio | *p* |
| 4 | PKM2 | 1.12 | 0.488 | 1.22 | 0.001 | 2.55 | 0.004 | 0.62 | 0.062 |
|  | ANXA2 | 1.54 | 0.371 | 3.54 | 0.001 | 2.44 | 0.012 | 0.69 | 0.942 |
|  | HSPB1 | 5.42 | 0.005 | 2.56 | 0.009 | 2.66 | 0.955 | 0.62 | 0.512 |
| 7 | PKM2 | 0.44 | 0.071 | 0.20 | 0.149 | 2.81 | 0.024 | 0.42 | 0.186 |
|  | ANXA2 | 0.00 | 0.000 | 0.06 | 0.007 | 4.63 | 0.003 | 0.19 | 0.006 |
| 14 | VIM | 0.76 | 0.015 | 0.12 | 0.029 | 0.56 | 0.001 | 0.23 | 0.224 |
|  | PKM2 | 0.50 | 0.012 | 0.19 | 0.004 | 1.28 | 0.088 | 0.33 | 0.868 |
| 21 | VIM | 4.78 | 0.000 | 2.89 | 0.007 | 1.40 | 0.394 | 0.76 | 0.056 |
|  | LMNA | 2.88 | 0.000 | 2.22 | 0.000 | 0.41 | 0.001 | 0.61 | 0.023 |
|  | ANXA1 | 0.89 | 0.435 | 0.58 | 0.016 | 0.49 | 0.001 | 0.11 | 0.001 |
