# Supplementary material for: Proteomics analysis of differentially expressed proteins in chicken trachea and kidney after infection with the highly virulent and attenuated coronavirus infectious bronchitis virus in vivo
Source: Proteome Sci. 2012 Mar 31;10:24. doi: 10.1186/1477-5956-10-24 (PMC3342233; doi:10.1186/1477-5956-10-24)
Supplement: Additional file 7 — Table S4 Comparison of the fold changes for protein abundance observed by 2-DIGE gel analysis and mRNA expression obtained by real-time RT-PCR in kidney tissues. [file 1477-5956-10-24-S7.DOC]

Table S4 Comparison of the fold changes for protein abundance observed by 2-DIGE gel analysis and mRNA expression obtained by real-time RT-PCR in kidney tissues

| dpi | Gene | P5-infected/control | | | | P115-infected/control | | | |
| --- | --- | --- | --- | --- | --- | --- | --- | --- | --- |
|  |  | 2-DIGE | | Real-time RT-PCR | | 2-DIGE | | Real-time RT-PCR | |
|  |  | ratio | *p* | ratio | *p* | ratio | *p* | ratio | *p* |
| 4 | MNSOD | 1.52 | 0.015 | 1.82 | 0.15 | 1.12 | 0.02 | 3.16 | 0.43 |
|  | PCK2 | 4.27 | 0.015 | 1.91 | 0.61 | 3.60 | 0.019 | 4.16 | 0.00 |
|  | MET24 | 0.72 | 0.00 | 1.27 | 0.05 | 1.09 | 0.07 | 1.91 | 0.03 |
| 7 | ANXA5 | 2.04 | 0.0035 | 0.77 | 0.00 | 1.10 | 0.75 | 0.62 | 0.01 |
|  | ACP1 | 6.34 | 0.05 | 0.34 | 0.00 | 0.74 | 0.16 | 0.62 | 0.07 |
| 14 | MET24 | 1.25 | 0.07 | 1.30 | 0.74 | 0.49 | 0.00 | 0.93 | 0.01 |
| 21 | APOA1 | 0.97 | 0.31 | 0.72 | 0.34 | 1.58 | 0.000043 | 0.31 | 0.00 |
|  | MET24 | 1.65 | 0.00037 | 0.83 | 0.01 | 1.87 | 0.0000094 | 1.49 | 0.63 |
