# Supplementary material for: Proteomics analysis of differentially expressed proteins in chicken trachea and kidney after infection with the highly virulent and attenuated coronavirus infectious bronchitis virus in vivo
Source: Proteome Sci. 2012 Mar 31;10:24. doi: 10.1186/1477-5956-10-24 (PMC3342233; doi:10.1186/1477-5956-10-24)
Supplement: Additional file 8 — Table S5 Experiment design of the different fluorescent dye labeling. [file 1477-5956-10-24-S8.DOC]

Table S5 Experiment design of the different fluorescent dye labeling

|  | Cy2 (internal standard) | Cy3 | Cy5 |
| --- | --- | --- | --- |
| *4 dpi* | | | |
| Gel-11 | 50 µg (25 µg each of samples kc11-13, ka11-13, kb11-13, Ka11*) | 50 µg kc11 | 50 µg ka13 |
| Gel-12 | 50 µg (25 µg each of samples kc11-13, ka11-13, kb11-13, Ka11*) | 50 µg kb13 | 50 µg ka12 |
| Gel-13 | 50 µg (25 µg each of samples kc11-13, ka11-13, kb11-13, Ka11*) | 50 µg ka11 | 50 µg kc13 |
| Gel-14 | 50 µg (25 µg each of samples kc11-13, ka11-13, kb11-13, Ka11*) | 50 µg kc12 | 50 µg kb11 |
| Gel-15 | 50 µg (25 µg each of samples kc11-13, ka11-13, kb11-13, Ka11*) | 50 µg kb12 | 50 µg ka11* |
| *7 dpi* | | | |
| Gel-21 | 50 µg (25 µg each of samples kc21-23, ka21-23, kb21-23, Ka21*) | 50 µg kc21 | 50 µg ka23 |
| Gel-22 | 50 µg (25 µg each of samples kc21-23, ka21-23, kb21-23, Ka21*) | 50 µg kb23 | 50 µg ka22 |
| Gel-23 | 50 µg (25 µg each of samples kc21-23, ka21-23, kb21-23, Ka21*) | 50 µg ka21 | 50 µg kc23 |
| Gel-24 | 50 µg (25 µg each of samples kc21-23, ka21-23, kb21-23, Ka21*) | 50 µg kc22 | 50 µg kb21 |
| Gel-25 | 50 µg (25 µg each of samples kc21-23, ka21-23, kb21-23, Ka21*) | 50 µg kb22 | 50 µg ka21* |
| *14 dpi* | | | |
| Gel-31 | 50 µg (25 µg each of samples kc31-33, ka31-33, kb31-33, Ka31*) | 50 µg kc31 | 50 µg ka33 |
| Gel-32 | 50 µg (25 µg each of samples kc31-33, ka31-33, kb31-33, Ka31*) | 50 µg kb33 | 50 µg ka32 |
| Gel-33 | 50 µg (25 µg each of samples kc31-33, ka31-33, kb31-33, Ka31*) | 50 µg ka31 | 50 µg kc33 |
| Gel-34 | 50 µg (25 µg each of samples kc31-33, ka31-33, kb31-33, Ka31*) | 50 µg kc32 | 50 µg kb31 |
| Gel-35 | 50 µg (25 µg each of samples kc31-33, ka31-33, kb31-33, Ka31*) | 50 µg kb32 | 50 µg ka31* |
| *21 dpi* | | | |
| Gel-41 | 50 µg (25 µg each of samples kc41-43, ka41-43, kb41-43, Ka41*) | 50 µg kc41 | 50 µg ka43 |
| Gel-42 | 50 µg (25 µg each of samples kc41-43, ka41-43, kb41-43, Ka41*) | 50 µg kb43 | 50 µg ka42 |
| Gel-43 | 50 µg (25 µg each of samples kc41-43, ka41-43, kb41-43, Ka41*) | 50 µg ka41 | 50 µg kc43 |
| Gel-44 | 50 µg (25 µg each of samples kc41-43, ka41-43, kb41-43, Ka41*) | 50 µg kc42 | 50 µg kb41 |
| Gel-45 | 50 µg (25 µg each of samples kc41-43, ka41-43, kb41-43, Ka41*) | 50 µg kb42 | 50 µg ka41* |

Note: The kidney protein samples of P_5_-infected group, P_115_-infected group and control group was indicated by ka, kb and kc. The first Arabic number indicated the days post infection, the second Arabic number indicated different biological individual.
