# Supplementary material for: Proteomics analysis of differentially expressed proteins in chicken trachea and kidney after infection with the highly virulent and attenuated coronavirus infectious bronchitis virus in vivo
Source: Proteome Sci. 2012 Mar 31;10:24. doi: 10.1186/1477-5956-10-24 (PMC3342233; doi:10.1186/1477-5956-10-24)
Supplement: Additional file 9 — Table S6 The primers of Real-time RT-PCR. [file 1477-5956-10-24-S9.DOC]

Table S6 The primers of Real-time RT-PCR

| Gene symbol | Gene accession No. | Forward primer sequence (5’-3’) | Reverse primer sequence (5’-3’) | Amplicon size (bp) |
| --- | --- | --- | --- | --- |
| MNSOD | AF329270 | GACCTGCCTTACGACTATGG | GCTGAAGCGACACCTGAG | 169 |
| PCK2 | NM_205470 | CGACATTGCGTGGATGAAG | CGTTGGTGAAGATGGTGTTG | 149 |
| ANXA5 | NM_001031538 | GGCTGGCACTGATGATGATACC | CCACCACAGAGGAGCAGGAG | 171 |
| ACP1 | NM_001039291 | AGTCTGTGCTCTTCGTTTGTCTG | CATGCAAGTCTGTCCTCGATAGTC | 173 |
| MET24 | XM_421871 | AGGATGGCTCGGTTGATC | TGGTGTAGGTGCTGATGG | 126 |
| APOA1 | NM_205525 | GCGAGATGTGGCTGAAGG | CTTGGCGGAGAACTGGTC | 107 |
| ANXA2 | NM_205351 | CTGTGATTGACTATGAACTGATTG | TTAACTTCCTTCTTGATGCTCTC | 196 |
| HSPB1 | NM_206906 | CTGGTGGTGAAGACTAAGGATAAC | GGGTGTATTTGCGGGTGAAG | 106 |
| VIM | NM_001048076 | GGATGTTGACAATGCCTCTC | TGCTGTTCCTGGAGTTGAG | 135 |
| LMNA | NM_205287 | CACCGCCCTCATCAACTC | CTTCGTCGTCCTCCTCATC | 101 |
| ANXA1 | NM_206906 | GGACAACCAGGAGCAGGAATG | TGGCTTCATCTACACCCTTTACAG | 134 |
| PKM2 | NM_205469 | TGCTCCACGGTTCCTACAAG | CAACACAGACAGACACCATAGAAG | 159 |
| 18S rRNA | FM165414 | CGGACAGGATTGACAGATTGAG | GCCAGAGTCTCGTTCGTTATC | 117 |
